# Data-driven modeling of supercritical CO₂ processing with optimized machine learning: prediction of clobetasol propionate solubility

**DOI:** 10.3389/fmed.2026.1954445

**Published:** 2026-09-07

**Authors:** Nidal H. Abu-Hamdeh, Mohammed N. Ajour

**Affiliations:** 1 Center of Research Excellence in Renewable Energy and Power Systems/Energy Efficiency Group, Department of Mechanical Engineering, Faculty of Engineering, King Abdulaziz University, Jeddah, Saudi Arabia; 2 Department of Electrical and Computer Engineering, Faculty of Engineering, King Abdulaziz University, Jeddah, Saudi Arabia

**Keywords:** computational analysis, drug nanoparticles, pharmaceutical engineering, solubility, supercritical solvent

## Abstract

Machine learning can be used to support data-driven modeling of supercritical CO₂ processing. The method of machine learning modeling is applied in this work for evaluation of small-molecule processing under supercritical conditions. As a necessary step, the solute solubility in the solvent is evaluated via different models. The purpose of this investigation is to develop models for the solubility of Clobetasol Propionate (CP) based on the two parameters of temperature and pressure as the model's features. Adaptive Boosting (AdaBoost) is utilized in this study on top of three core models: Decision Tree (DT), Lasso, and Gaussian Process Regression (GPR). The models are tuned through the firefly algorithm (FA) to determine their unknown coefficients. The final models are called FB-DT (FA-optimized Boosted DT), FB-GPR (FA-optimized Boosted GPR), and FB-LASSO (FA-optimized Boosted LASSO) and have R^2^ scores of 0.934, 0.977, and 0.813, respectively, for fitting the solubility data of CP. Accordingly, FB-GPR is the most accurate model, with an RMSE of 1.11 × 10⁻^2^. The developed models demonstrate great performance in estimating CP solubility in supercritical CO₂ under different temperature and pressure conditions.

## 1 Introduction

Solubility of Active Pharmaceutical Ingredients (APIs) is an important parameter to judge about the efficiency of the drug and the dose needed to be taken by patients. Moreover, solubility is required as the key parameter in designing some unit operations in pharmaceutical engineering and manufacture of solid-dosage formulations where solution-based processing is conducted (1–3). For the crystallization of small-molecules APIs, the solubility is critical to be determined as function of temperature for design of process. Moreover, some models based on thermodynamics can be developed along with experimental measurements to estimate API solubility in a wide range of operations without the need for expensive and time-consuming experimentation (4–7).

For preparation of nanomedicines, solubility measurements and estimation are of great importance. In nanomedicine preparation, solubility is used to determine whether the process is viable to be adopted for nanonization of the considered API. One of the main implications of the nanonization is determination of APIs solubility for treatment of drug particles using supercritical solvent which is mainly carbon dioxide. Different experimental methods have been proposed for measuring solubility of APIs, among which the method of gravimetric is the most commonly used method (8–10). On the other hand, some research has been carried out on correlations of drug solubility to non-linear models to predict the solubility values at various conditions. The methods of semi-empirical, machine learning, thermodynamics, and molecular modeling are the main computational approaches for estimation of API solubility in supercritical solvents (11, 12).

The term known as machine learning (ML) refers to a group of techniques that enable computing devices to learn from data without actually being directly programmed. The goal of ML is to develop meta-programs that are able to interpret experimental data via learning the pattern (13, 14). We used the Adaboost method to enhance the performance of GPR, Decision Tree, and Lasso regression models on prediction of the solubility of Clobetasol Propionate (CP) as the model API.

Boosting is an ensemble technique that combines weak models to form a strong one capable of making accurate predictions. A weak (base) model developed on the train data is the first step in boosting approach. When modest changes in data create big changes in the predictive model, a weak classifier learning algorithm is weak. The new model emphasizes or lends more weight to cases that were incorrectly predicted in the previous round in the next iteration (15, 16).

Adaptive Boosting (AdaBoost) is a widely applied ensemble-learning technique that can be integrated with different base-learning algorithms to enhance predictive performance. It sequentially constructs a collection of weak learners, with each successive learner placing greater emphasis on observations that were predicted poorly in the preceding stage. The individual predictions are subsequently aggregated through a performance-based weighted combination, producing a stronger and more accurate final model.

In this study, the Decision Tree (DT) regression algorithm was employed as the base learner within the AdaBoost framework to improve predictive performance. A Decision Tree constructs a hierarchical structure by recursively partitioning the dataset according to feature values. At each decision node, the algorithm selects the optimal feature and threshold to split the data into two subsets, thereby minimizing prediction error (17, 18).

The non-parametric Bayesian GPR (Gaussian process regression) model can be utilized in situations involving both exploitation and exploration. One of the many benefits of using this software is that it can give reliable responses based on the characteristics that are input. Utilizing an infinite number of input attributes is supposed to make it possible to display a wide variety of correlations between the features that are fed in and the results that are generated. Bayesian inference is a method that can be used to determine the level of complexity in models (19–22).

Here we used, for the first time, three efficient machine learning models for determination of Clobetasol Propionate (CP) drug solubility at various circumstances to analyze its conditions for preparation of nanosized particles. The employed models of machine learning here are: Decision Tree (DT), Lasso, and Gaussian Process Regression (GPR). Thus, the novelty is in application of data-driven models and combination for this drug in improving the solubility estimation.

## 2 Dataset of pharmaceutical solubility

The dataset used in this investigation is sourced from a published article (23), and includes two inputs: temperature in Kelvin units and pressure in MPa units. This data set contains 45 rows of data collected at five temperatures and nine pressure levels. The values have been selected for keeping the solvent (CO_2_) at the supercritical zone for all experiments and analyses. Their work reported the experimental measurements for solubility of Clobetasol Propionate (CP) in CO_2_ at the supercritical pressure and temperature zone. Lahiq et al. (24) used this data for implementing some ML models to correlate the solubility, and the method has been used in this work for model development as well as further analysis using new data-driven models.

**Figure 1:**
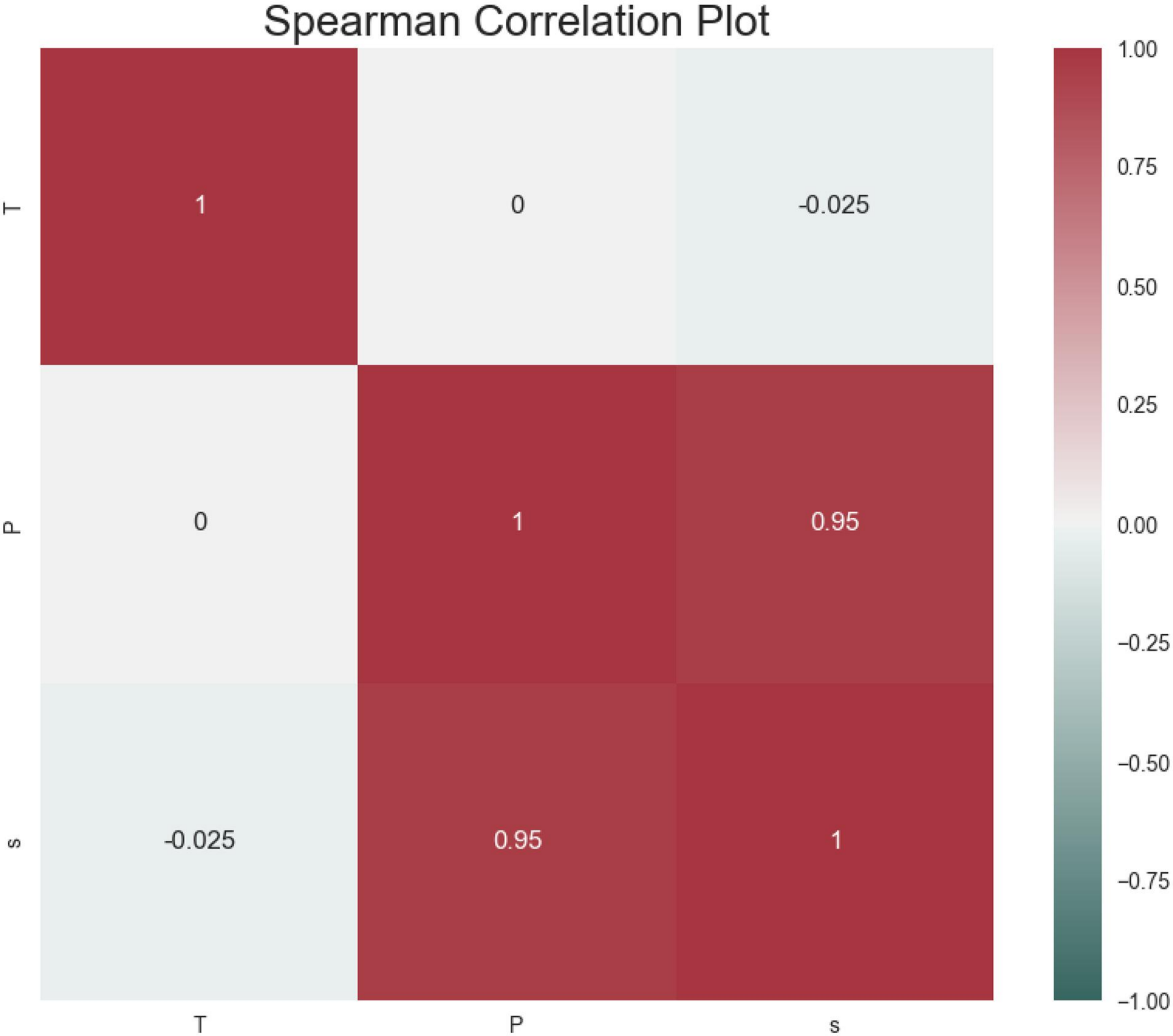
Spearman correlation plot for CP drug solubility analysis.

Figure 1 also depicts the Spearman correlation heat map of the dataset (23).

## 3 Modeling methodology

### 3.1 Firefly algorithm

The Firefly Algorithm (FA), originally introduced by Yang (25), is a population-based optimization method that translates the light-signaling behavior of fireflies into a numerical search procedure. A firefly represents one feasible candidate solution, and its position contains the values of the decision variables. The quality of that solution is represented by its brightness, which is calculated from the objective-function value. Thus, for a maximization problem, a brighter firefly corresponds to a better solution; for a minimization problem, the objective value is converted into an appropriate fitness measure before brightness is assigned. FA is commonly described through three idealized rules: fireflies are treated as unisex, attraction is stronger toward brighter individuals, and perceived brightness decreases with distance because light is absorbed by the surrounding medium. Consequently, a comparatively weak solution is updated in the direction of a better solution, while the best candidate continues to explore through a controlled random displacement.

For two fireflies, *i* and *j*, the distance-dependent attractiveness can be expressed as:

$$\beta (r_{ij})=\beta_{0}\exp(-\gamma r_{ij}^{2}),$$

where *β*₀ denotes attractiveness when the distance is zero, *γ* is the light-absorption coefficient, and *r*ᵢ_j_ is the distance between the two candidate solutions. When firefly *j* is brighter than firefly *i*, the latter is updated according to

$$x_{i}^{\;t+1}=x_{i}^{\;t}+\beta_{0}\exp(-\gamma r_{ij}^{2})(x_{j}^{\;t}-x_{i}^{\;t})+\alpha_{t}\varepsilon_{i}^{\;t},$$

where **x**ᵢᵗ and **x**_j_ᵗ are their positions at iteration *t*, *α*ₜ determines the intensity of random exploration, and ***ε***ᵢᵗ is a random vector. The second term guides the search toward a promising region and therefore supports exploitation, whereas the last term introduces diversity and enables exploration of regions that are not represented by the current best candidates. If no brighter firefly is available, movement is governed primarily by this random component (25, 26).

### 3.2 GPR model

GPR presupposes that subsequent observations have knowledge about one another. For an unspecified objective function, GPR assumes that the response at every given place follows a Gaussian distribution. To be specific, it is expected that the outputs of a function will follow a multivariate Gaussian distribution if numerous input-output data points are collected. This is due to the fact that the Gaussian distribution was first designed to characterize the connection between input features and output targets. So, for instance, the GPR prediction context states the following about the response of function *f* (21):

y=f(x)+∈

The symbol ∈ stands for the Gaussian noise distribution, which is a common statistical model (21):

$$\in N(0,\sigma^{2})$$

The f(x) is supposed to follow the GP declared by mean (m(x)) and the covariance ($k(x,x^{\prime })$) in Gaussian Process Regression (27, 28).

### 3.3 Lasso model

Lasso regression is a feature selection and prediction methodology that was developed by Tibshirani (29). This method is useful for keeping constraints on the parameters that reduce coefficients toward zero for the purpose of variable reduction. Obtaining the subset of characteristics that results in the least amount of error in prediction for a response variable is the objective of the lasso regression technique. For Lasso regression, the sum of the square of error is denoted by (29):

SSELasso=∑(y−y^)2+λ∑|β|

Here, *y* is the observed value, y^ is the estimated value, λ is the parameter that determines the amount of shrinkage, and β is the regression coefficients.

### 3.4 Decision tree

The primary objective of the Decision Tree (DT) learning algorithm is to construct a predictive model capable of accurately estimating a target response variable or assigning the correct class label to unseen data samples. To achieve this goal, the DT algorithm utilizes a hierarchical tree-based structure, where internal nodes represent decision rules based on selected input features, branches indicate the outcomes of these decisions, and leaf nodes correspond to the final predicted values or class labels (30, 31). During the tree construction process, selecting the most informative feature for each split is essential for improving model performance.

Entropy is a statistical measure used to quantify the uncertainty, disorder, or randomness present within a dataset. For a set of observations consisting of different outcomes, where each observation has an associated probability, the entropy can be calculated using the following expression (32, 33):

$$\mathrm{Entropy}=\;-\sum p_{i}\;log_{2}(\;p_{i})$$

### 3.5 AdaBoost method

AdaBoost (Adaptive Boosting) is a widely used ensemble learning technique in ML due to its ability to improve prediction accuracy compared with individual weak learning algorithms. AdaBoost was implemented in this study as a regression ensemble following the general principles of AdaBoost. Unlike conventional AdaBoost regression, which commonly employs shallow decision trees as its base estimators, the present framework used Gaussian Process Regression (GPR) and Least Absolute Shrinkage and Selection Operator (LASSO) regression as alternative weak learners. The resulting hybrid models are referred to as AdaBoost-GPR and AdaBoost-LASSO, respectively. In each hybrid model, boosting was applied sequentially so that every newly fitted GPR or LASSO learner focused more strongly on observations that had been predicted poorly by the preceding learners.

**Figure 2:**
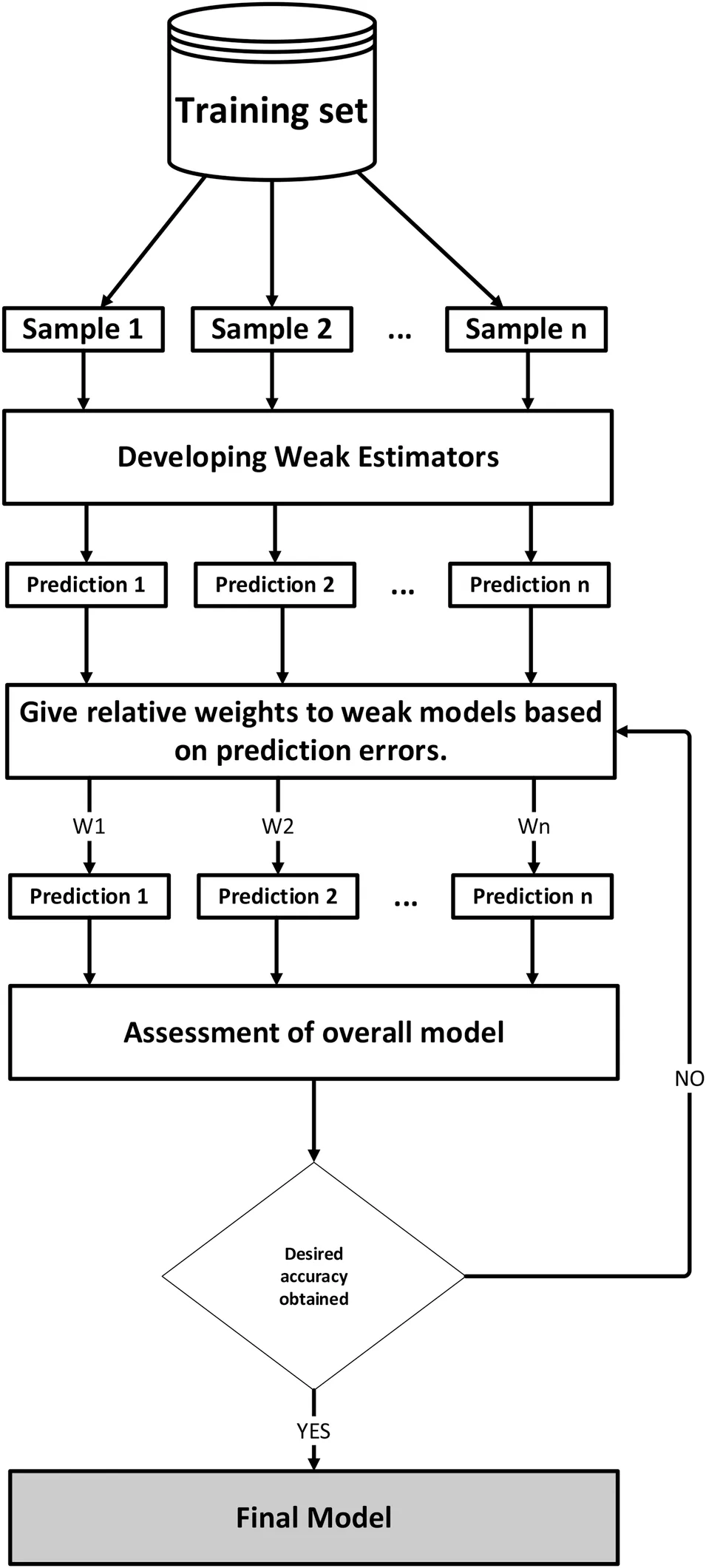
The workflow of adaBoost ensemble method designed in this work for modeling.

Finally, the outputs of all individual learners are combined through a weighted summation, resulting in a reliable ensemble model with improved generalization capability (34, 35). Figure 2 displays the conceptual representation of AdaBoost model designed for modeling in this study.

## 4 Results and discussions

**Figure 3:**
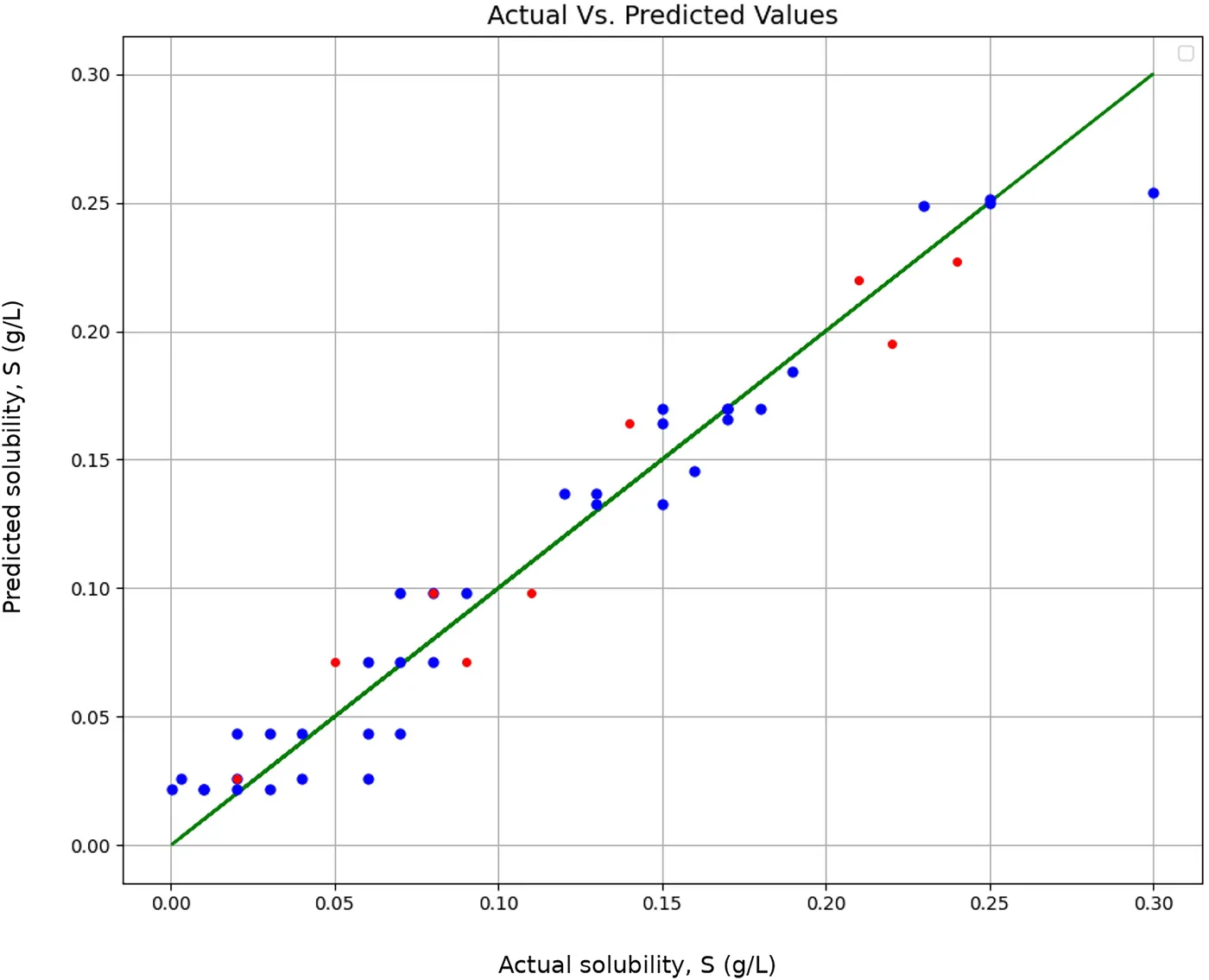
Expected vs. estimated values (FB-DT model).

**Figure 4:**
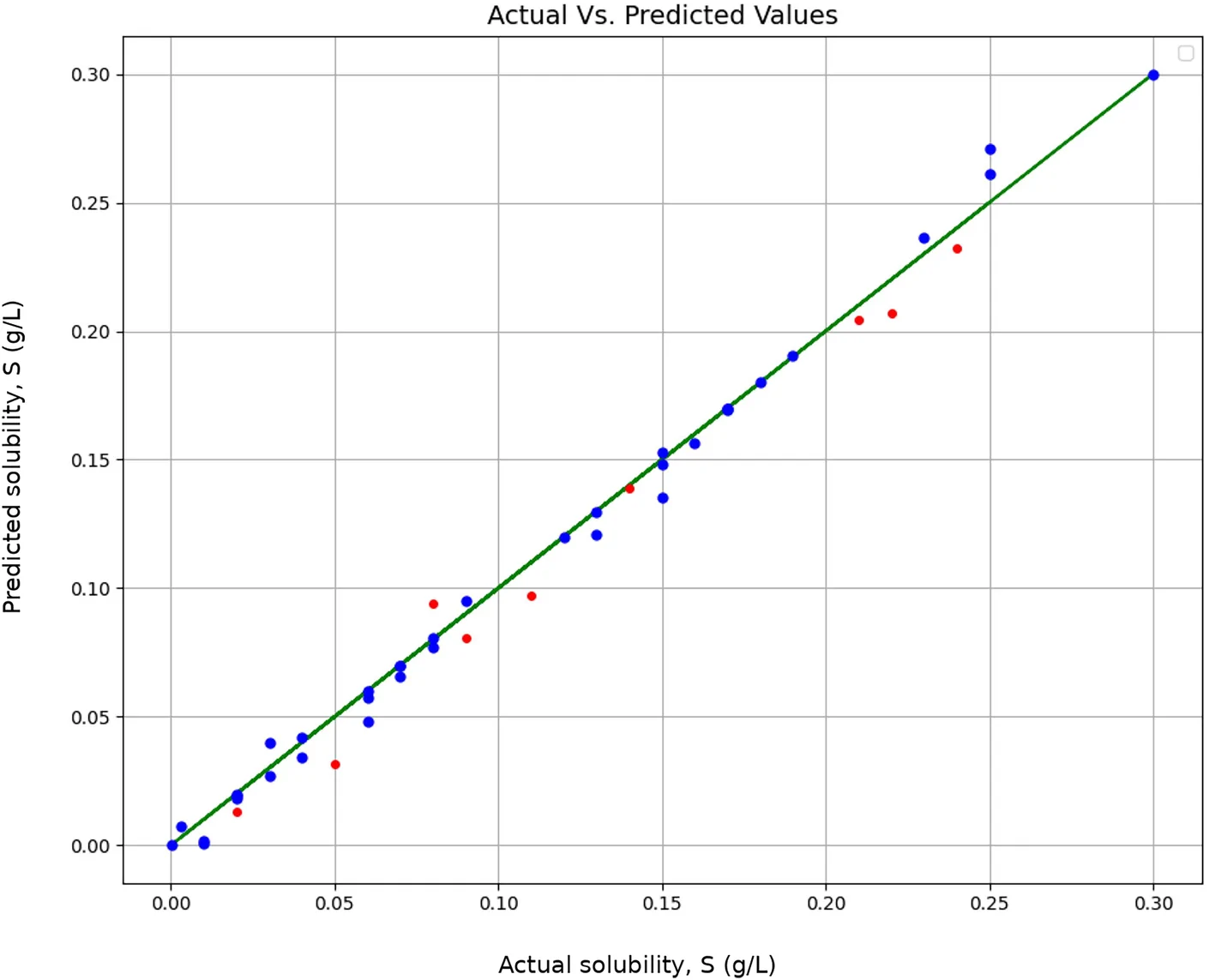
Expected vs. estimated values (FB-GPR model).

**Figure 5:**
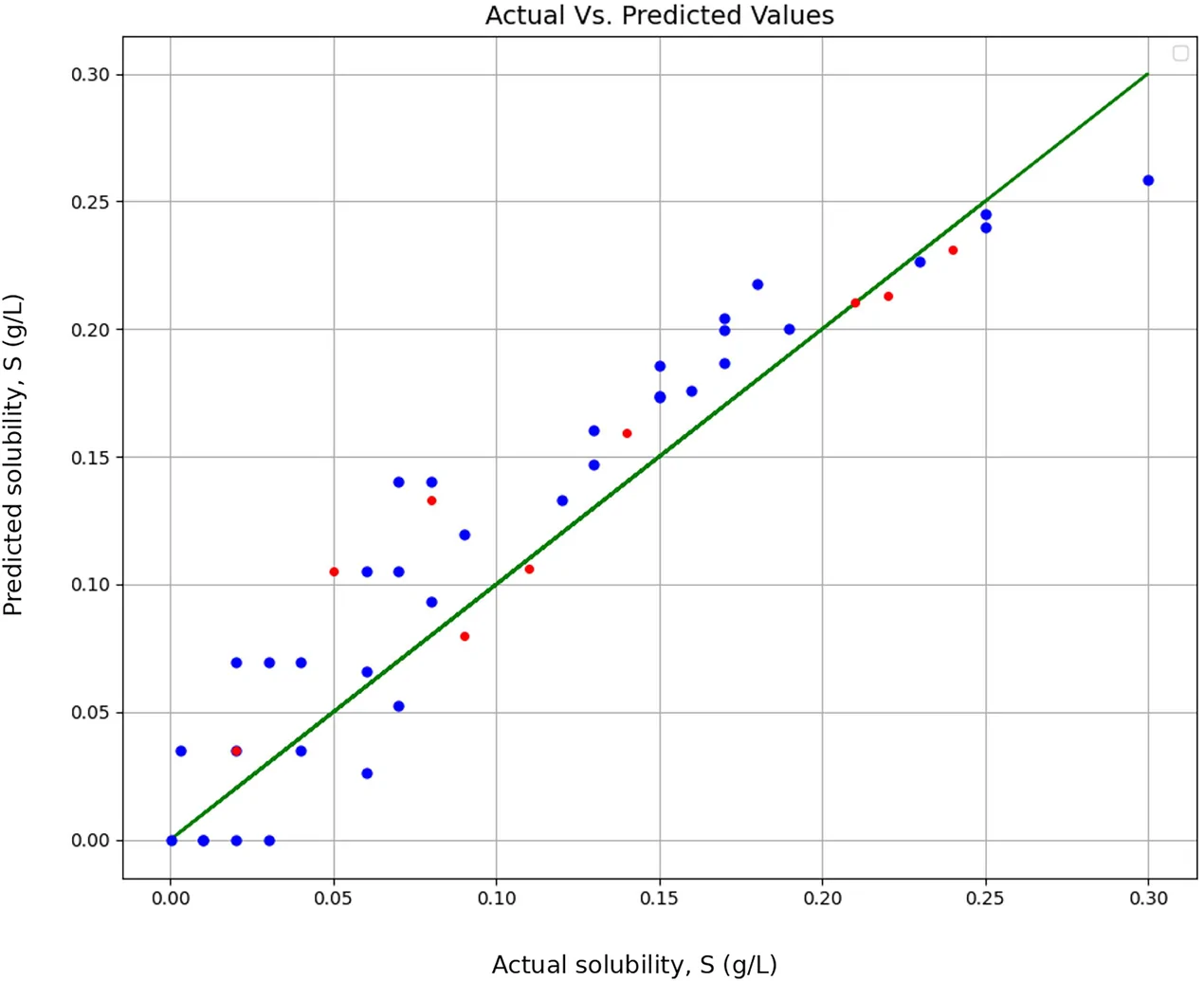
Expected vs. estimated values (FB-LASSO model).

The hybrid models that were discussed before were tweaked, and their tuned hyper-parameters calculated for improving the precision of models. After that, the ML designed models that were examined and their respective coefficients of determination (R^2^) and errors are presented in Table 1. Figures 3–5 demonstrate a visual comparison of the models by comparing the observed values to the values predicted by the three models. All of these facts and data confirm that the FB-GPR model is the most appropriate among these three models in terms of accuracy and generality for determination of CP solubility.

**Table 1:** Accuracy and performance of final model.

Models	R^2^ Score	RMSE	Max Error
FB-DT	0.934	1.76 × 10^−2^	1.84 × 10^−2^
FB-GPR	0.977	1.11 × 10^−2^	2.50 × 10^−2^
FB-LASSO	0.813	2.73 × 10^−2^	5.53 × 10^−2^

**Figure 6:**
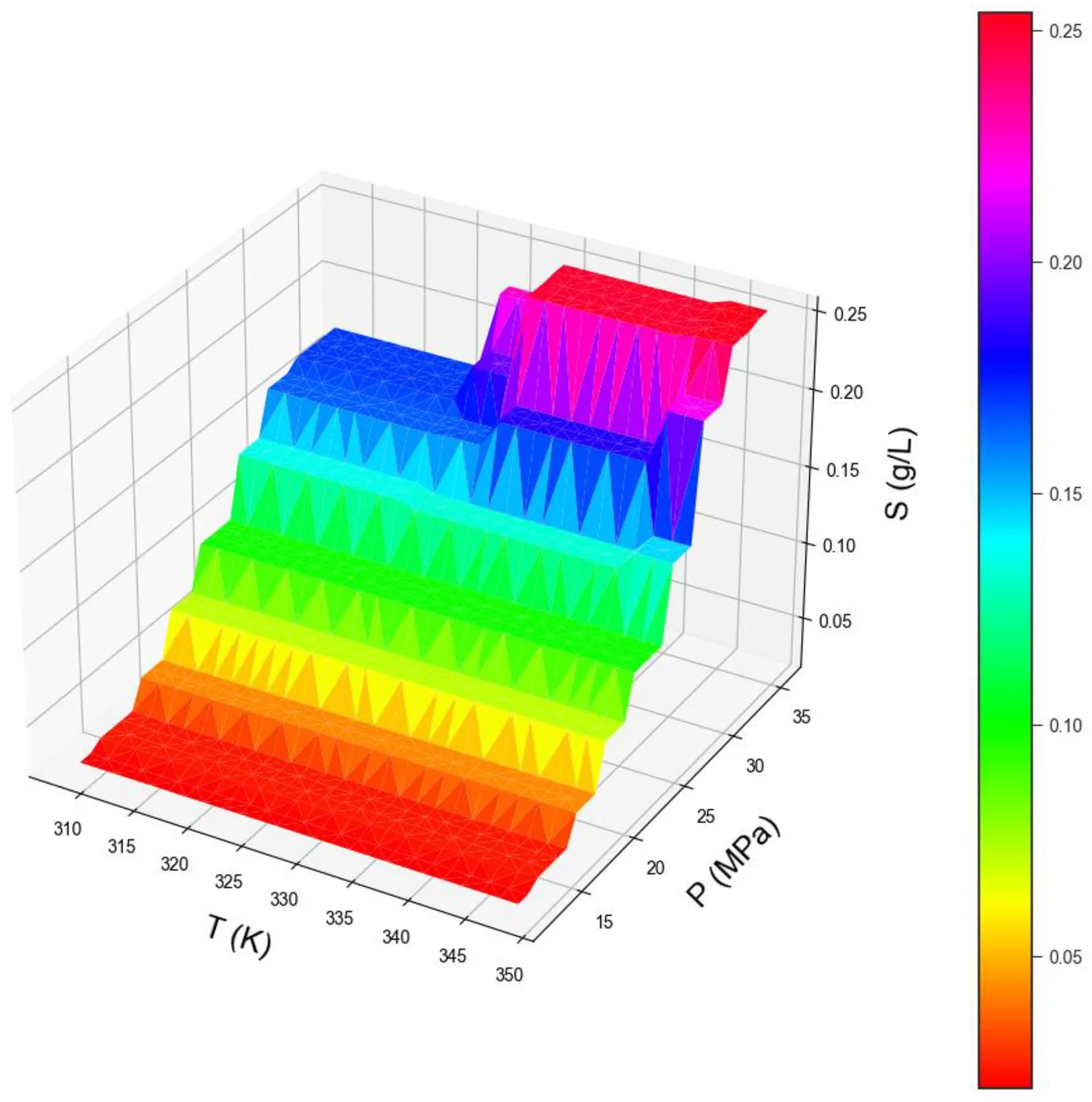
3D surface of estimator (FB-DT model).

**Figure 7:**
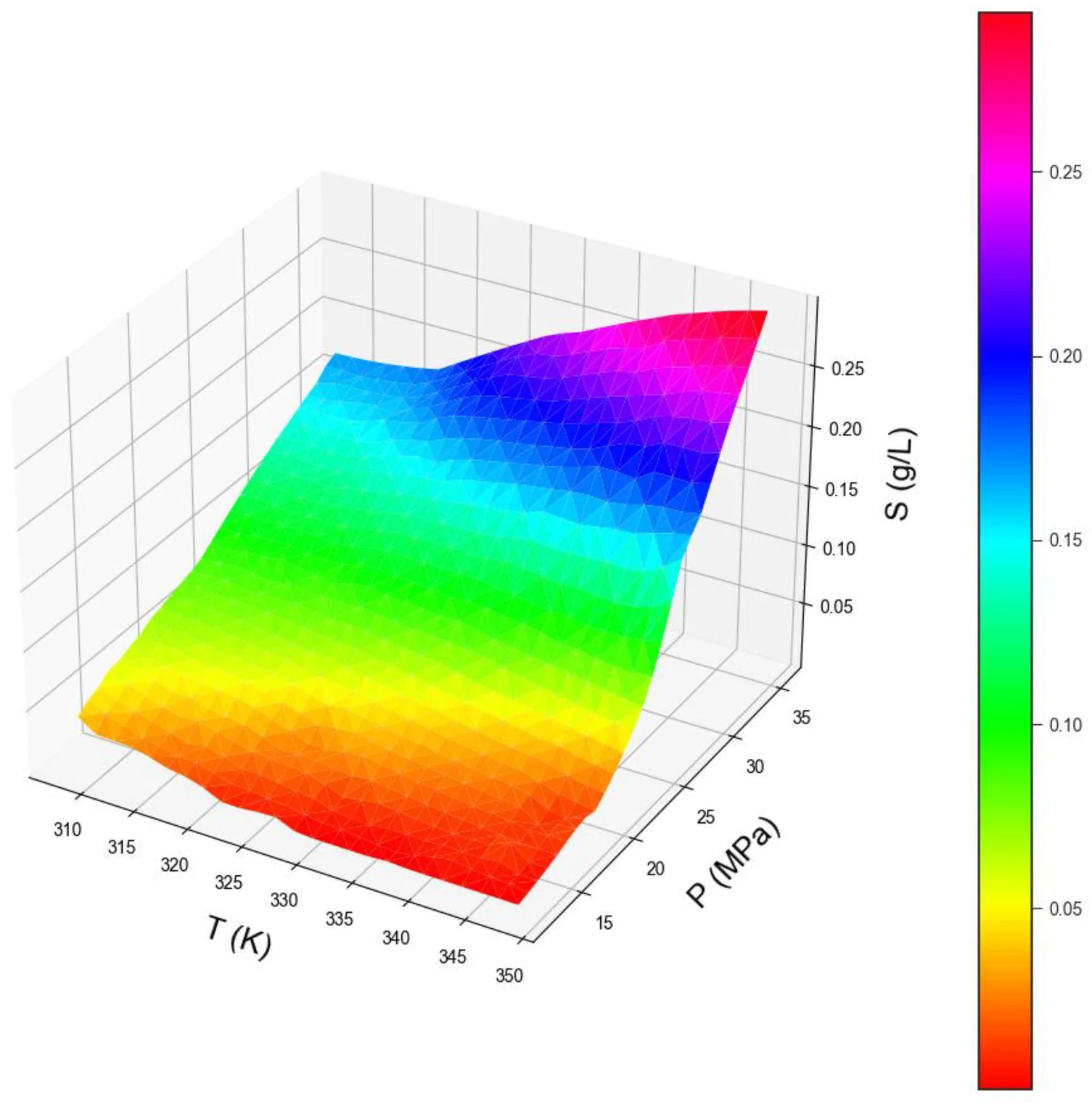
3D surface of estimator (FB-GPR model).

**Figure 8:**
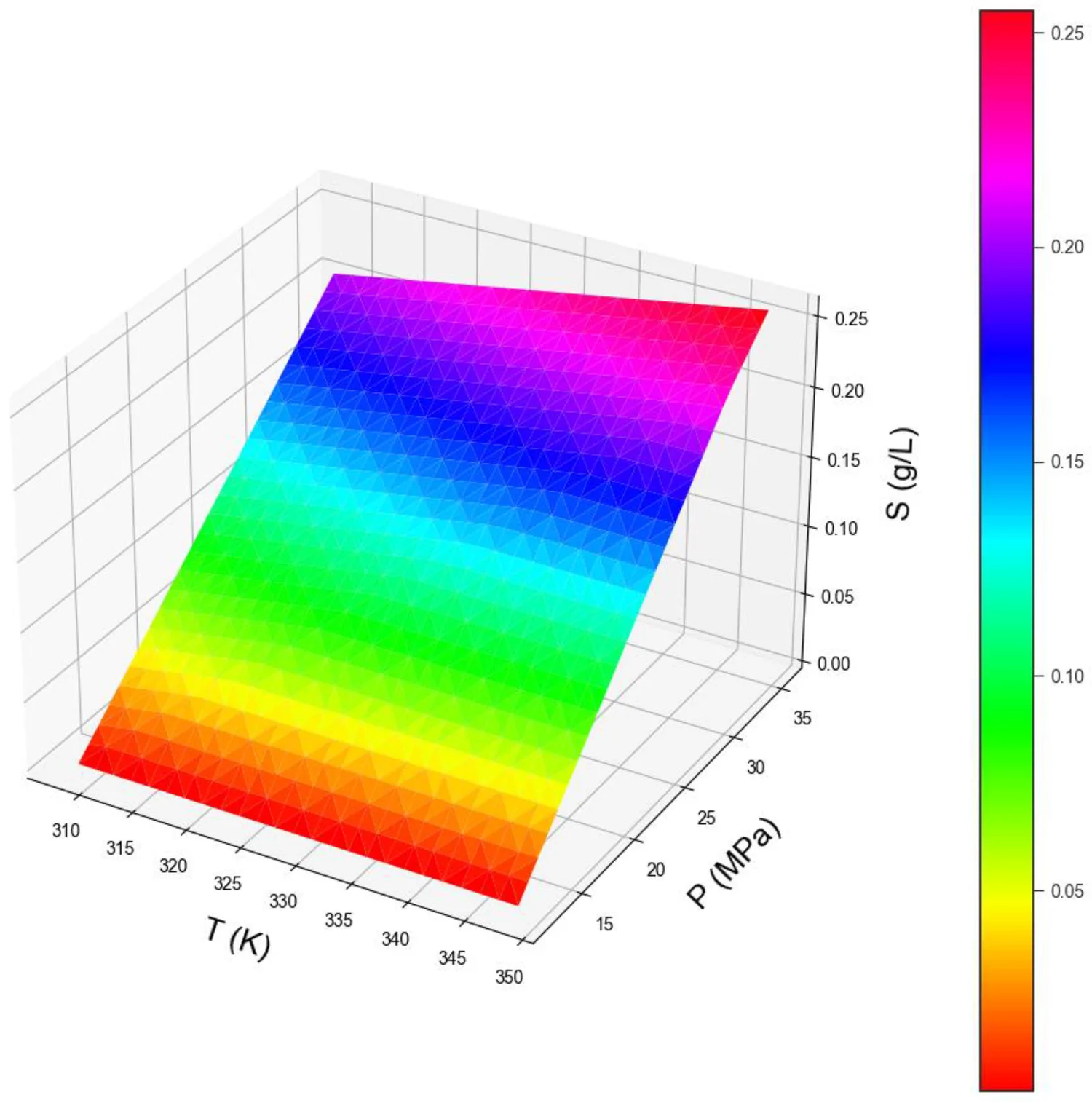
3D surface of estimator (FB-LASSO model).

**Figure 9:**
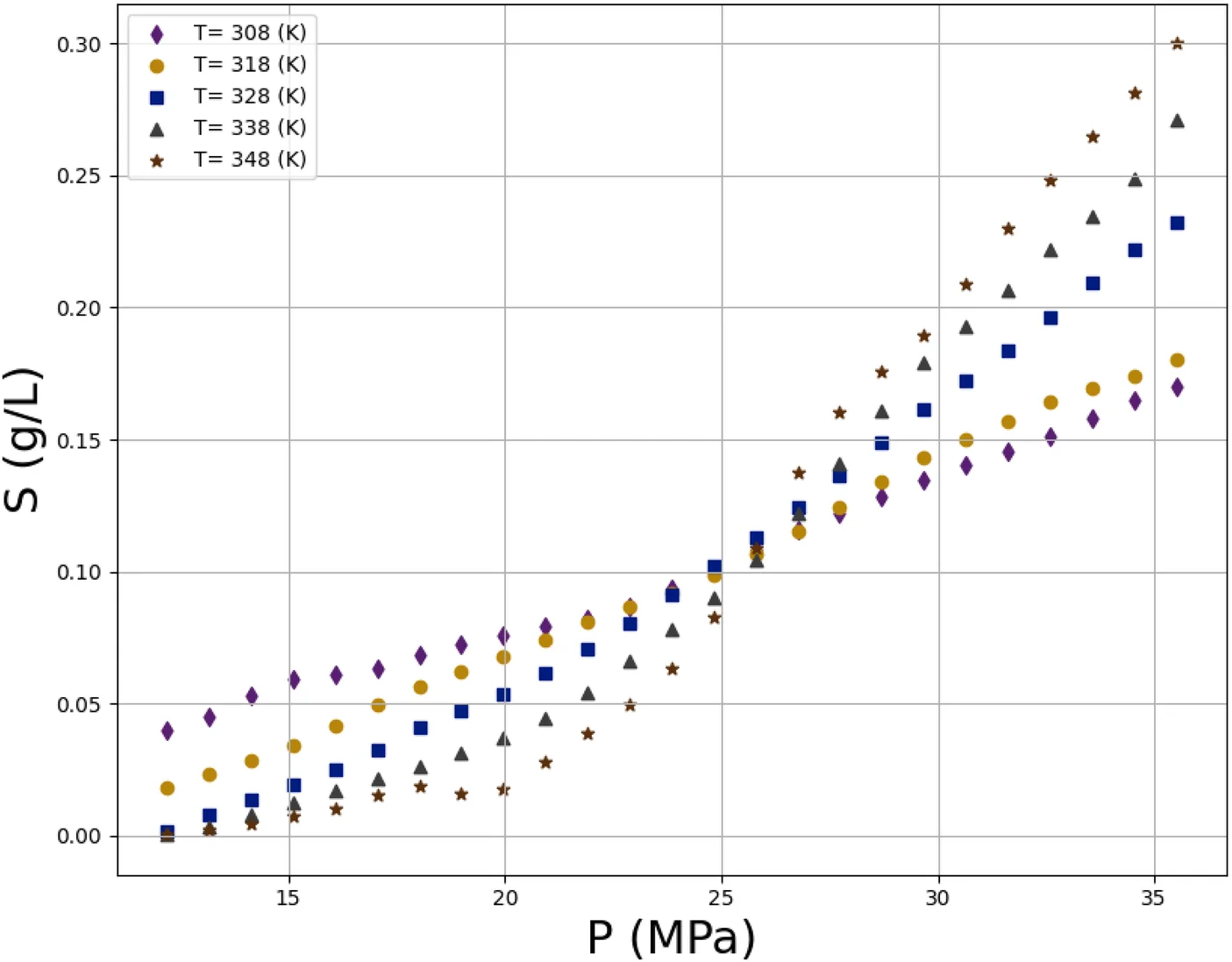
Tendencies on *P* variable.

**Figure 10:**
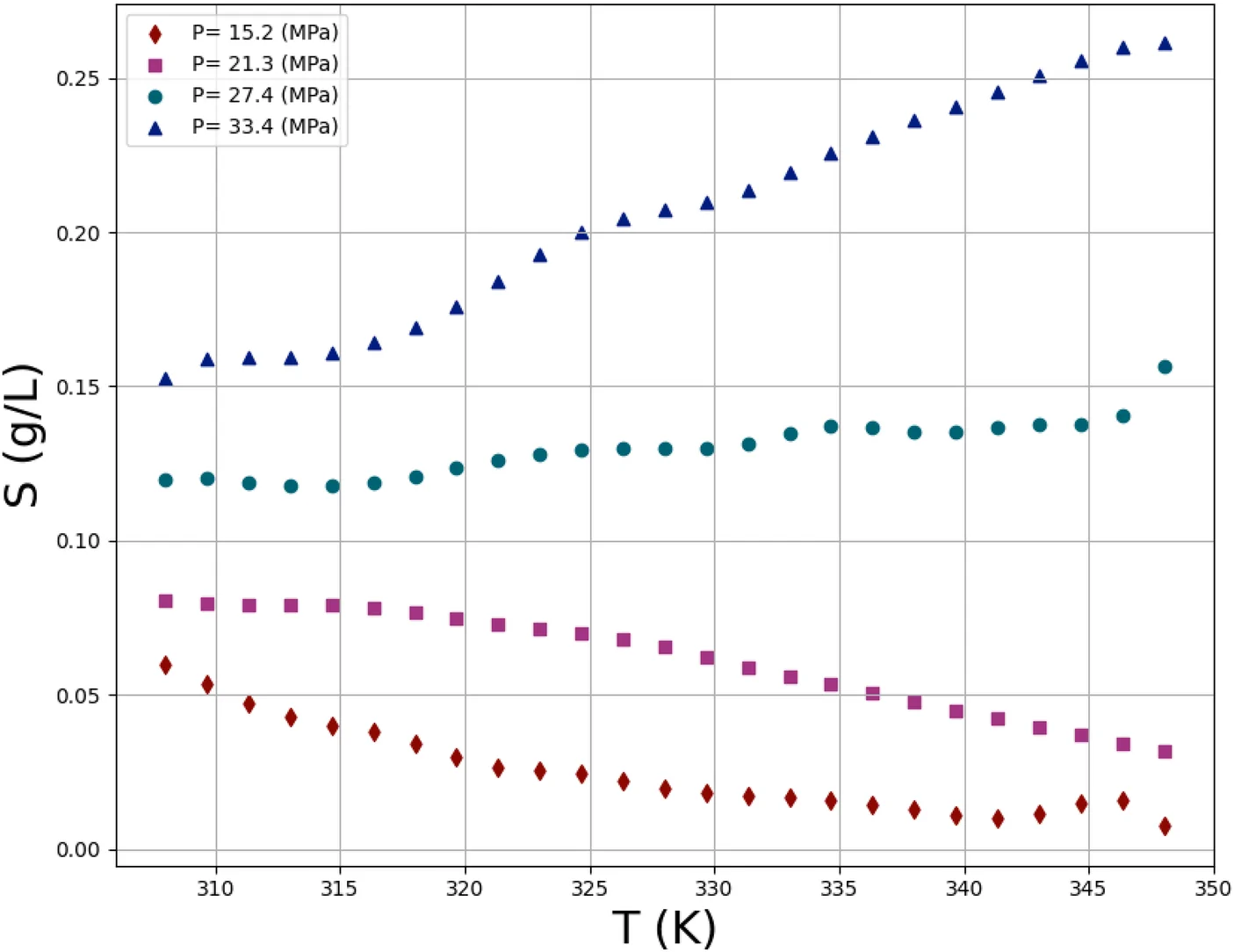
Tendencies on T variable.

Furthermore, Figures 6–8 present the three-dimensional response surfaces generated by the developed models. The predicted surfaces exhibit similar overall trends, although slight differences can be observed in terms of smoothness and variation patterns. Figures 9, 10 provide 2D representations of the influence of input variables on the response of the FB-GPR model, which demonstrated the best predictive performance among the investigated models (36). The results indicate that pressure has a significant effect on the predicted solubility values of CP, highlighting its important role in controlling the solubility behavior of the system. Unlike liquid solvents, the solvent used in this study is supercritical gas which behaves pseudo-liquid solvent and pressure enhance can compress it and accommodate more drug molecules which in turn increases the drug solubility. Previous studies revealed some similar behavior for API solubility in supercritical CO_2_ (24, 36–38). Moreover, the results are in agreement with the model's findings of Lahiq et al. (24) and Liang et al. (36) for CP.

Figure 9 illustrates the combined effects of pressure and temperature on API solubility in the solvent, i.e., supercritical CO₂. At all investigated temperatures, solubility increases with pressure because compression raises the density and solvent strength of supercritical CO₂, thereby enhancing drug–solvent interactions in the mixture. Nevertheless, the influence of temperature depends strongly on pressure. In the lower-pressure region (approximately 12–22 MPa), solubility decreases as temperature increases; for example, the values at 308 K are higher than those at 348 K. Under these conditions, the reduction in CO₂ density caused by heating dominates over the increase in the drug's vapor pressure. A crossover region occurs at approximately 23–27 MPa, where the solubility is comparatively insensitive to temperature and the isotherms intersect. Beyond this region, the temperature effect is reversed: higher temperatures produce markedly greater solubility because the enhancement in solute vapor pressure becomes more influential than the accompanying decrease in CO₂ density. Consequently, at 36 MPa, solubility increases from approximately 0.17 g/L at 308 K to 0.30 g/L at 348 K. This crossover behavior is characteristic of solid–supercritical-fluid equilibria and demonstrates that pressure and temperature must be optimized simultaneously to maximize drug solubility in supercritical CO₂.

## 5 Conclusion

The aim of this study is to create models for the solubility of Clobetasol Propionate (CP) based on two parameters: temperature and pressure. The results would be essential for design of supercritical processing in production of solid dosage nanomedicine with improved solubility. In this study, Adaptive Boosting (AdaBoost) is used with fundamental models: DT, Lasso, and GPR. The firefly algorithm is used to tune the models. The resulting models are known as FB-DT (FA optimized Boosted DT), FB-GPR (FA optimized Boosted GPR), and FB-LASSO (FA optimized Boosted LASSO), and have R^2^ values of 0.934, 0.977, and 0.813, respectively. As a result, the FB-GPR is the most accurate model, with an RMSE error rate of 1.11 × 10^−2^. It should be pointed that the main limitation is the small data size for building robust models, and more data would create more robust models which can be generalized in this context.

## Data Availability

The original contributions presented in the study are included in the article/Supplementary Material, further inquiries can be directed to the corresponding author.
